# Atg5 Regulates Selective Autophagy of the Parental Macronucleus during *Tetrahymena* Sexual Reproduction

**DOI:** 10.3390/cells10113071

**Published:** 2021-11-08

**Authors:** Tao Bo, Yu Kang, Ya Liu, Jing Xu, Wei Wang

**Affiliations:** 1 Key Laboratory of Chemical Biology and Molecular Engineering of Ministry of Education, Institute of Biotechnology, Shanxi University, Taiyuan 030006, China; botao@sxu.edu.cn (T.B.); 201923002007@email.sxu.edu.cn (Y.K.); 201723002012@email.sxu.edu.cn (Y.L.); xujing@sxu.edu.cn (J.X.); 2 College of Life Sciences, Shanxi University, Taiyuan 030006, China

**Keywords:** autophagy-related protein 5, parental macronucleus, programmed nuclear degradation, *Tetrahymena thermophila*

## Abstract

Nuclear autophagy is an important selective autophagy process. The selective autophagy of sexual development micronuclei (MICs) and the programmed nuclear degradation of parental macronucleus (paMAC) occur during sexual reproduction in *Tetrahymena thermophila*. The molecular regulatory mechanism of nuclear selective autophagy is unclear. In this study, the autophagy-related protein Atg5 was identified from *T. thermophila*. Atg5 was localized in the cytoplasm in the early sexual-development stage and was localized in the paMAC in the late sexual-development stage. During this stage, the degradation of meiotic products of MIC was delayed in *atg5i* mutants. Furthermore, paMAC was abnormally enlarged and delayed or failed to degrade. The expression level and lipidation of Atg8.2 significantly decreased in the mutants. All these results indicated that Atg5 was involved in the regulation of the selective autophagy of paMAC by regulating Atg8.2 in *Tetrahymena.*

## 1. Introduction

Autophagy is an essential and highly conserved process of intracellular component degradation. Harmful substances, misfolded proteins, and damaged organelles are separated and wrapped into the autophagosomes, which are transported into lysosomes and degrade the cargoes [1,2,3,4]. This process is essential for maintaining cell physiological homeostasis, including cell growth, development, repair, and survival, as well as responding to starvation or other environmental stresses [5]. Disorders in autophagy regulation are related to many human diseases, such as neurodegenerative diseases, metabolic disorders, and cancer [6,7,8]. Organelle degradation is regulated based on cell function and energy requirements [9]. Nuclear autophagy is related to diamond-blackfan anemia, cancer, and various human diseases [10,11]. In yeast, two types of nuclear autophagy exists, namely, piecemeal microautophagy of the nucleus that is regulated by the connection between the nucleus and vacuoles mediated by the interaction of Vac8p and Nvj1p [12,13,14], and the selective degradation of part of the nucleus mediated by Atg39. The interaction between Atg39 and Atg8 promotes the formation of the autophagosome membrane that encloses part of the nucleus to complete degradation [15]. In mammalian nuclear envelopathies, the nucleus is degraded by autophagy when the nucleus is destroyed or partially separated into the cytoplasm [16]. The entire nucleus is degraded by autophagy in cultured murine seminal-vesicle epithelial cells [17]. The differentiation of keratinocytes leads to the formation of the stratum corneum during keratinization, and nuclear dissolution occurs through autophagy in this process. Failure to degrade the nucleus in the stratum corneum leads to parakeratosis, which is a characteristic of psoriasis. The expression levels of the autophagy proteins LC3, WIPI1, and ULK1 decrease in the epidermal parakeratotic area of patients. However, the mechanisms that control nucleophagy in mammals and how to establish selectivity remain poorly understood [18].

Atg proteins play a critical role in modulating autophagic processes and activity. The core Atg proteins can be grouped into different functional units: the Atg1/ULK complex, phosphatidylinositol 3-kinase (PI3K) complex, Atg2-Atg18/WIPI4 complex, Atg9 vesicle, Atg12-Atg5 conjugation system, and Atg8/LC3 conjugation system [19]. In the yeast autophagy system, Atg5 is one of the core proteins of autophagy [20]. Atg5 forms a complex with ubiquitin-like protein Atg12 and Atg16 and is located on the pre-autophagosomal structure and the outer surface of isolation membrane during autophagy. E1-like enzyme Atg7 activates Atg12 and transfers it to Atg10. Atg12 then covalently bonds with Atg5. After the Atg5-Atg12 conjugate associates with Atg16 [21], Atg12-Atg5-Atg16 complex mediates the activation of E2-like enzyme Atg3 and then regulates the lipidation of Atg8. This complex is involved in determining the localization of lipidated Atg8 [22]. Atg5 is also associated with mitochondrial quality control after oxidative damage, and it negatively regulates the innate antiviral immune response by directly binding to retinoic acid receptor responder protein 3 and mitochondrial antiviral signaling protein. Atg5 is also involved in the regulation of lymphocyte development and proliferation, the presentation of MHC II antigen, and the differentiation and apoptosis of adipocytes [20]. Furthermore, Atg5 binds to selective autophagy receptors (SARs) through the Atg8 interaction motif of the yeast SARs (Atg19 and Atg34) and LC3 interaction region of human SARs (p62/SQSTM1, NDP52, and OPTN) [23,24]. The interaction between the Atg8 interaction motif of SARs and Atg8 or Atg5 is mutually exclusive. How the Atg12-Atg5-Atg16 complex recognizes cargo receptors in vivo remains unclear. The function of ATG5 in selective autophagy requires further investigation [25].

The ciliate *Tetrahymena* is a free-living ciliate group that is ubiquitous in water ecosystems worldwide [26]. *Tetrahymena thermophila* displays nuclear dimorphism containning germline micronucleus (MIC) and somatic macronucleus (MAC). The MIC is the germline, the storage of genetic information for the sexual progeny. The MAC is transcriptionally active in the vegetative cells and is thus considered the somatic nucleus [27]. *T. thermophila* has been used as a model organism in the studies of genetics, cell biology, and toxicology [28]. During the sexual reproduction of *Tetrahymena*, the programmed degradation of parental macronucleus (paMAC) is performed by autophagy with the development of new macronucleus [29]. The programmed nuclear degradation (PND) of paMAC during the sexual reproduction of *Tetrahymena* provides an ideal model for studying the molecular-regulation mechanism of nuclear autophagy [29]. The PND of paMAC is also known as gigantic nuclear macroautophagy [15]. The autophagy-related proteins Atg8-2p and Atg8-65p are localized on the paMAC membrane, and numerous small autophagosomes and lysosomes approach and engulf the paMAC under the regulation of TtVps34, Atg8-2p, and Atg8-65p. Lysosomes and small autophagosomes release endogenous substances, such as apoptosis-inducing factor (AIF) and mitochondrial nuclease 1, resulting in the acidification of paMAC and complete DNA degradation [13,30,31,32,33]. AIF in the cytoplasm can also be enriched in the paMAC under the regulation of Ran1 [29]. Atg8s regulate the formation of autophagosomes and fusion between the autophagosomes and nuclear membrane. Vps34 is involved in the regulation of lysosome recruitment [31,34,35]. Atg5 forms a complex with Atg12 and Atg16 to regulate the lipidation and localization of Atg8 in yeast and mammalian cells. However, we have identified only Atg16 homologs (TTHERM_00721780 and TTHERM_00294550) and have not yet obtained Atg12 homologs in *Tetrahymena*. In this context, it is not clear how the selective autophagy of paMAC is regulated. Whether Atg5 is involved in regulating the PND process and, if so, how it functions is also unknown during PND. In the present study, Atg5 was localized in the paMAC during the PND stage, and paMAC was abnormally enlarged and delayed or failed to degrade after knocking down *ATG5.* In addition, the degradation of meiotic products of MIC was delayed in the *atg5i* mutants. Atg5 mediated the PND of the paMAC by regulating the function of Atg8.2.

## 2. Materials and Methods

### 2.1. Cell Culture and Conjugation

The wild-type (WT) *Tetrahymena* strains B2086 (II) and CU428 (mpr1-1/mpr1-1 [VII, mp-s]) were obtained from the National *Tetrahymena* Stock Center (Available online: http://tetrahymena.vet.cornell.edu/index.html, accessed on 1 November 2021). Cells were cultured in 1× SPP medium (1% proteose peptone, 0.1% yeast extract, 0.2% glucose, and 0.003% sequestrene) at 30 °C. Conjugation was induced by mixing equal amounts of B2086 and CU428 cells in 10 mM Tris-HCl (pH 7.5) at 30 °C [36].

### 2.2. Bioinformatic Analysis

Orthologous *ATG5* genes from fungi and mammals were searched using the program blastp algorithm in the *Tetrahymena* Genome Database (http://ciliate.org, accessed on 1 November 2021). The potential proteins interacting with Atg5 were investigated by STRING (version 11.0, ELIXIR Hub, Cambridge, London, UK) [37,38]. The conserved domain within the protein sequences was analyzed using the Simple Modular Architecture Research Tool (SMART) database (http://smart.embl-heidelberg.de/, accessed on 1 November 2021) [39]. Multiple sequence alignment was conducted using Clustal X2 (Version 2.0, Science Foundation Ireland, Dublin, Ireland) and default parameters were used for the analysis [40]. The alignment figure was drawn using Jalview (version 2.10.3, The Barton Group, University of Dundee, Scotland, UK) [41]. A phylogenetic tree was constructed through the neighbor-joining method using MEGA software (version:7.0.14, Mega Limited, Auckland, New Zealand) [42].

### 2.3. Construction of HA-ATG5 and HA-ATG8.2 Strains

*ATG5* and *ATG8.2* were amplified from genomic DNA by PCR using primers as shown in Table S1. Then, *ATG5* and *ATG8.2* were inserted into the pXS75 vector separately. HA-*ATG5* and HA-*ATG8.2* expression levels were controlled by the *MTT1* promoter under Cd^2+^ induction. pXS-HA-*ATG5* and pXS-HA-*ATG8.2* were digested with *Sac I/Xho I* and subsequently introduced into the B2086 and CU428 strains using the biolistic particle-transformation system GJ-1000 (SCIENTZ, Ningbo, Zhejiang, China), as previously described [43,44]. Transformants were selected on the basis of resistance to paromomycin and were identified by PCR with the *MTT1*-FW/*MTT1*-RV primer set (Table S1).

### 2.4. ATG5 Knockdown by RNA Interference

To create the *ATG5* knockdown construct, a 500-bp fragment of the *ATG5* ORF was amplified from genomic DNA using PCR primers as shown in Table S1 and then cloned into the RNA interference (RNAi) hairpin vector pSMC1hpNEO (gift from Josef Loidl, University of Vienna, Vienna, Austria) to create a hairpin expression cassette. The recombinant plasmid pSMC1hpNEO-*ATG5* was digested with *Blp* I and subsequently introduced into the B2086 and CU428 strains by using a biolistic particle-transformation system GJ-1000 (SCIENTZ, Ningbo, Zhejiang, China). Cells were selected on the basis of resistance to cycloheximide as previously described [45]. In all cases, RNAi was induced by adding 0.1 μg/mL CdCl_2_ to cells carrying the hairpin construct.

### 2.5. Labeling of Autophagosomal Structures and Lysosomes

Autophagic vacuoles, lysosome, and nuclei of living cells were labeled with the autofluorescent marker monodansylcadaverine (MDC) (Sigma–Aldrich, St. Louis, MO, USA, Cat. No. 30432), Lyso-ID dye-like Lysotracker Red (LTR) (Beyotime Biotechnology, Shanghai, China, Cat. No. C1046), and Hoechst 33342 (Beyotime Biotechnology, Shanghai, China, Cat. No. C1029), respectively, as previously described [31]. For photography, cells were anesthetized with 15 mM NiCl_2_ (Sigma–Aldrich, St. Louis, MO, USA, Cat. No. N6136), and digital images were acquired with an Olympus FV1000 laser scanning confocal microscope (Olympus Corporation, Tokyo, Japan).

### 2.6. Indirect Immunofluorescence Analysis

To observe the localization patterns of HA-Atg5 and HA-Atg8, cells were treated as previously described [36]. Mating cells were fixed overnight with 5 mL of Lavdowsky’s fixative (ethanol/formalin/acetic acid/water = 50:10:1:39) at 4 °C and immobilized on cover glasses coated with poly-L-lysine (Sigma–Aldrich). The fixed cells were washed with PBS and PBST (0.05% Triton X-100 or 0.1% Tween-20) three times for 10 min each time. Then, the cells were incubated with a 1:500 dilution of rabbit anti-HA antibodies (Covance, Berkeley, CA, USA) or 1:500 dilution of anti-Atg8 antibodies (Proteintech Group, Inc., 11010-1-AP) in a blocking solution, followed by 1:1000 fluorescein isothiocyanate (FITC)-conjugated goat anti-rabbit IgG in a blocking solution. DNA was subsequently stained with 1 μg/mL 4′,6-diamidine-2-phenylindole dihydrochloride (DAPI) (Roche Company, Beijing, China) in PBS. Digital images were captured with a Delta Vision Elite deconvolution microscope (Applied Precision/GE Healthcare, API company, Rockville, MD, USA) or Olympus FV1000 laser scanning confocal microscope and were processed using Adobe Photoshop (version:14.0, Adobe Company, San Jose, CA, USA).

### 2.7. Western Blot Analysis

Whole-cell proteins from 2.5 × 10^6^ cells were extracted and separated by 12% sodium dodecyl sulfate-polyacrylamide gel electrophoresis and then transferred onto PVDF membranes. Blots were incubated with a 1:500 dilution of rabbit anti-HA antibody in blocking solution (5% milk and 0.1% Tween 20 in PBS) and then visualized by incubation with a 1:1000 dilution of HRP-conjugated anti-rabbit IgG antibody (Zymed Labs Inc., South San Francisco, CA, USA) in blocking solution. Finally, the blots were reacted with Western Blot Chemiluminescence Reagent (NEN Life Science, Boston, MA, USA). Visualization was achieved with a SuperSignal chemiluminescence detection system (Pierce, Rockford, IL, USA).

### 2.8. Statistical Analysis

Student’s *t* test was used for statistical analysis with the SPSS statistical software package (Version 23.0, IBM SPSS Statistics Company, Armonk, New York, NY, USA). One asterisk (*) indicates *p* < 0.05, and two asterisks (**) indicate *p* < 0.01.

## 3. Results

### 3.1. Characterization of ATG5 in Tetrahymena

**Figure 1:**
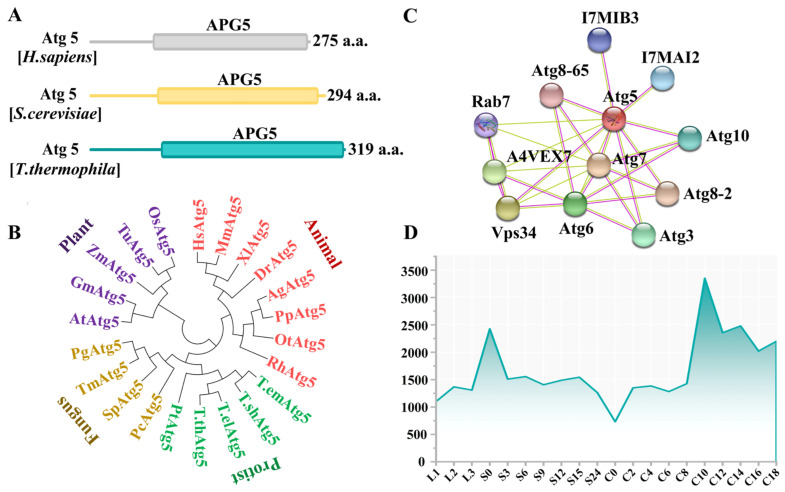
Characterization of Atg5 in *Tetrahymena thermophila.* (**A**) Schematic of the functional domain of the autophagy-related protein Atg5. Analysis was performed in the SMART database; (**B**) Phylogenetic tree of Atg5. Phylogenetic trees were constructed based on the amino acid sequences of APG5 domain from different species. HsAtg5, *Homo sapiens* Atg5 (AGC52703.1); MmAtg5, *Mus musculus* Atg5 (NP_444299.1); XlAtg5, *Xenopus laevis* Atg5 (AAH71093.1); DrAtg5, *Danio rerio* Atg5 (NP_991181.2); AgAtg5, *Anoplophora glabripennis* Atg5 (XP_018579453.1); PpAtg5, *Photinus pyralis* Atg5 (XP_031333397.1); OtAtg5, *Onthophagus taurus* Atg5 (XP_022904887.1); RhAtg5, *Rhipicephalus haemaphysaloides* Atg5 (QHA24496.1); *T.empidokyrea* Atg5, *Tetrahymena empidokyrea* Atg5 (TEPIDO00117960); *T.shanghaiensis* Atg5, *Tetrahymena shanghaiensis* Atg5 (TSHANG00053680); *T. elliotti* Atg5, *Tetrahymena elliotti* Atg5 (TELLIO00184010); *T. thermophile* Atg5, *Tetrahymena thermophila* Atg5 (XP_001023196.1); PtAtg5, *Paramecium tetraurelia* Atg5 (XP_001439074.1); PcAtg5, *Phytophthora cinnamomi* Atg5 (KAG6623658.1); SpAtg5, *Schizosaccharomyces pombe* Atg5 (NP_596427.1); TmAtg5, *Tuber magnatum* Atg5 (PWW76226.1); PgAtg5, *Pyricularia grisea* Atg5 (ABO93146.1); AtAtg5, *Arabidopsis thaliana* Atg5 (NP_197231.1); GmAtg5, *Glycine max* Atg5 (CAJ31277.1); ZmAtg5, *Zea mays* Atg5 (NP_001105827.1); TuAtg5, *Triticum urartu* Atg5 (EMS63970.1); OsAtg5, *Oryza sativa Japonica Group* Atg5 (XP_015627449.1); (**C**) Analysis of potential proteins interacting with Atg5 in *T. thermophila* using the STRING database. Each node represents a protein; (**D**) Expression pattern of the *ATG5* gene from *T. thermophila* Functional Genomics Database (http://tfgd.ihb.ac.cn, accessed on 1 November 2021). L, growing cells; S, starvation; C, conjugation.

**Figure 2:**
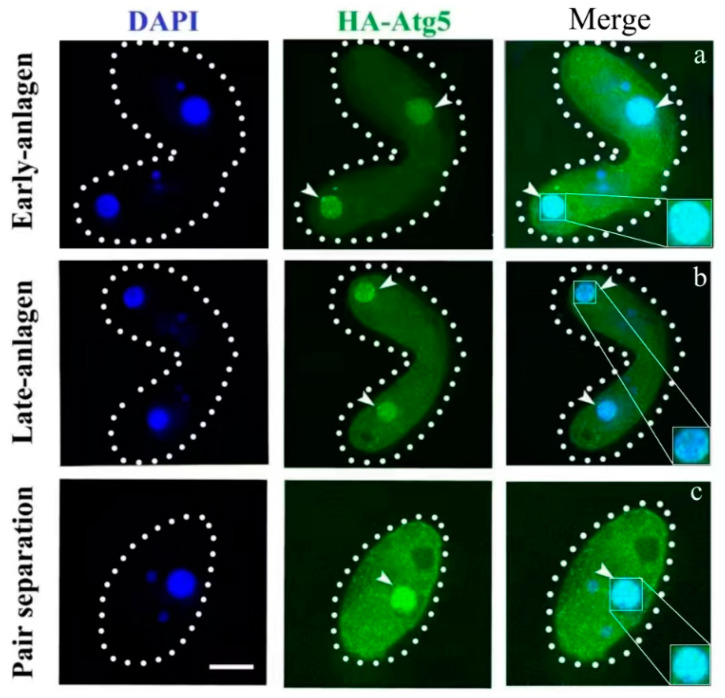
Localization of HA-Atg5 during the sexual reproduction of *Tetrahymena thermophila*. Cells collected at 6, 8, and 12 h after mixing were fixed and processed for immunofluorescence staining with anti-HA primary and FITC-conjugated secondary antibodies. Cellular nuclei were stained with DAPI to visualize DNA. (**a**) cells at the early-anlagen stage; (**b**) cells at the late-anlagen stage; (**c**) cells at the pair separation stage. Dashed circle represents the cell outline of *Tetrahymena*. The white arrows point to the paMAC to be degraded. The white box shows a sharp enlargement of the paMAC. Fluorescent images were taken with a DeltaVision deconvolution microscope. Scale bar, 10 µm.

Atg5 (TTHERM_00494030) was identified in the *Tetrahymena* genome database (http://www.ciliate.org, accessed on 1 November 2021) through homologous sequence alignments with *ATG5* from human and yeast. *ATG5* is 960 bp, has no introns, and encodes 319 amino acids (Figure 1A). Phylogenetic analysis showed that Atg5s were evolutionarily conservative (Figure 1B). The interacting factors of Atg5 were identified using the STRING online tool, including Atg3, Atg6, Atg7, Atg8, Atg10, and Vps34 (Figure 1C). Atg8 regulated the formation of autophagosomes and the fusion of autophagosomes with paMAC to form a huge autophagic structure. Loss of TtVps34 activity prevented autophagosome formation on the paMAC, and this nucleus escaped from the lysosomal pathway [31]. *ATG5* was expressed in the growth, starvation, and sexual-reproduction stages, and the expression level of *ATG5* was the highest at 10 h after conjugation (Figure 1D). To study the function of Atg5 during PND, HA-tagged Atg5 was expressed under the control of the *MTT1* promoter (Figure S2), which was induced by 0.2 μg/mL cadmium 2 h after conjugation. HA-Atg5 was localized in the cytoplasm in the early stage of sexual reproduction and began to accumulate on the paMAC from the anlagen stage (Figure S1 and Figure 2a). Following the development of mating cells, Atg5 was localized around paMAC until paMAC was completely degraded (Figure 2b,c). This result indicated that Atg5 could be involved in the regulation of PND of paMAC in *Tetrahymena*.

### 3.2. ATG5 Knockdown Inhibites the PND of paMAC

**Figure 3:**
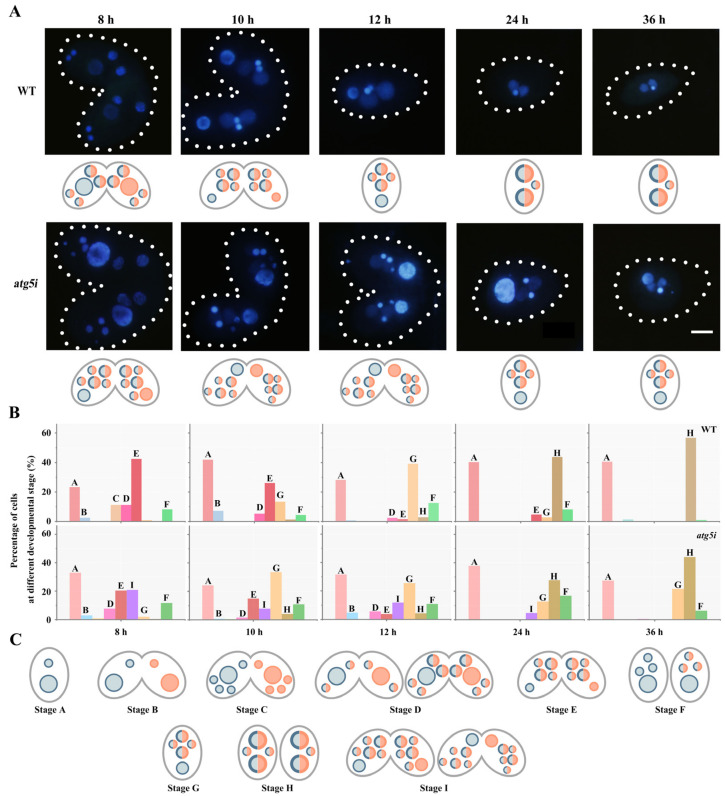
*ATG5* knockdown hindered programmed degradation of micronucleus meiosis products and the paMAC. (**A**): Nuclear development morphology in WT and *atg5i* cells during the PND stage. Dashed circle represents the cell outline of *Tetrahymena.* The image was taken with a laser scanning confocal microscopy, scale bar: 10 μm. (**B**): Percentage of different developmental stages during the sexual-reproduction stage (*n* > 200) in the wild-type and *atg5i* cell lines. Different capital letters (A–I) correspond to the different stages of cells in (**C**). (**C**): Schematic of sexual reproduction of cells in the wild-type and *atg5i* cell lines.

To analyze the function of *ATG5* during PND, *ATG5* knockdown mutants were constructed by RNAi (Figure S3A). The efficiency of RNAi of *ATG5* was verified by Western blotting. Atg5 disappeared under cadmium induction in the *atg5i* mutants (Figure S3B). Compared with WT cell lines, the paired cells developed normally in the first 6 h after conjugation in the *atg5i* mutants. At 8 h of conjugation, 42.6% of the WT paired cells developed into the anlagen stage, which contained two new MICs and two developed new macronuclei (MACs). By contrast, 39.8% of the *atg5i* mutant paired cells developed to the anlagen stage. However, the degradation of meiotic products was delayed in the mutants (Figure 3). At 10 h during sexual reproduction, paMAC migrated to the bottom of the cell in the WT. By contrast, paMAC failed to migrate from the anterior or middle of the cytoplasm to the posterior region in 12.7% of the *atg5i* mutants (Figure 3). Furthermore, 21.5% of *atg5i* mutants retained the paMAC at 36 h of sexual reproduction (Figure 3B,C). This result indicated that Atg5 was involved in regulating the PND of paMAC.

**Figure 4:**
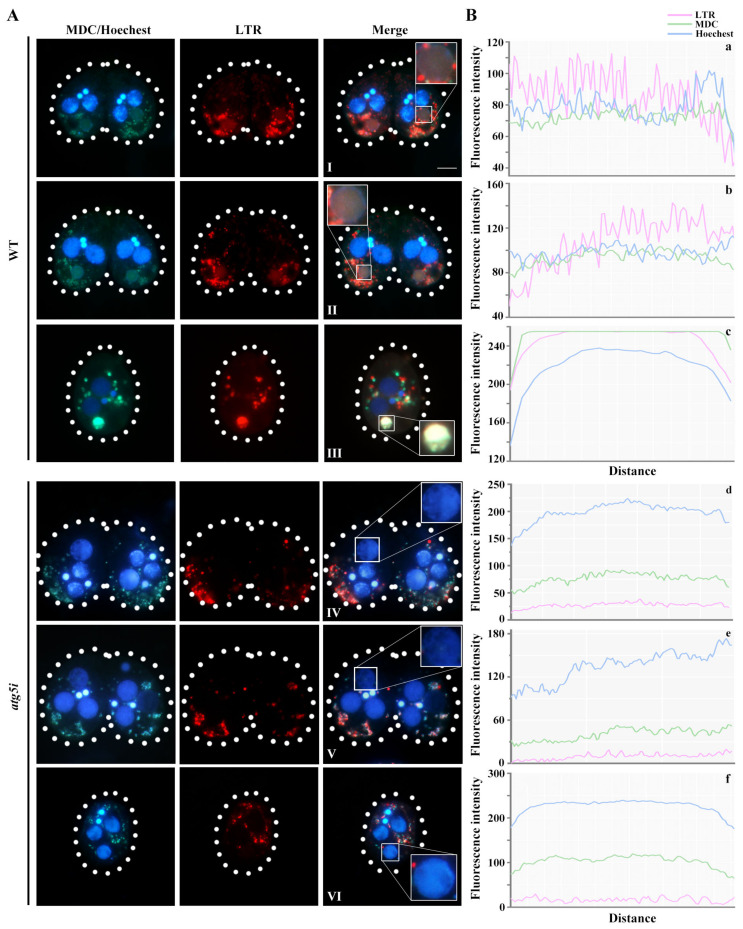
paMAC failed to acidify during PND in *atg5i* mutants. (**A**): Conjugating cells at 8 (I,IV), 10 (II,V), and 12 h (III,VI) were stained with MDC, LTR, and Hoechest in WT and *atg5i* cell lines. Dashed circle represents the cell outline of *Tetrahymena*. Scale, 20 μm; (**B**): The fluorescence intensities of the three probes were analyzed in the region of the selected nucleus (white box) in the merged diagram (left panel) with Image J (version:1.8.0, National Institutes of Health, Bethesda, MD, USA), corresponding to the a–f.

**Figure 5:**
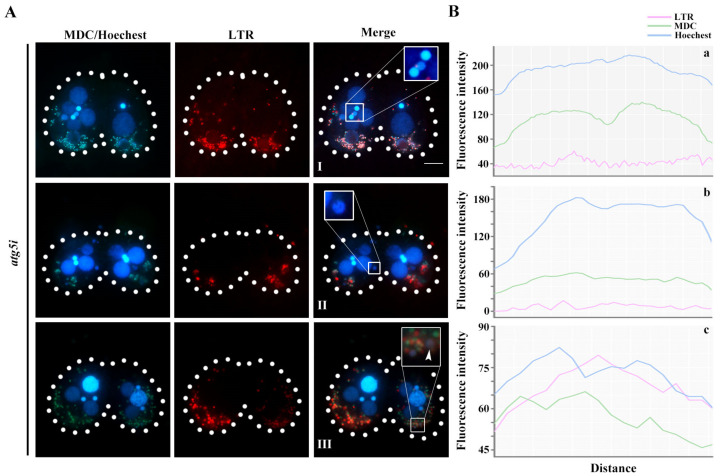
The acidification and degradation of meiotic products of MIC were delayed in *atg5i* cells. (**A**): Conjugating cells at 10 (I), 12 (II) and 24 h (III) were stained with MDC, LTR, and Hoechest in *atg5i* cell lines. The white arrows point to the acidifying meiotic products. Dashed circle represents the cell outline of *Tetrahymena*. scale, 20 μm; (**B**): The fluorescence intensities of the three probes were analyzed in the region of the selected nucleus (white box) in the merged diagram (left panel) with Image J, corresponding to the a–c.

**Figure 6:**
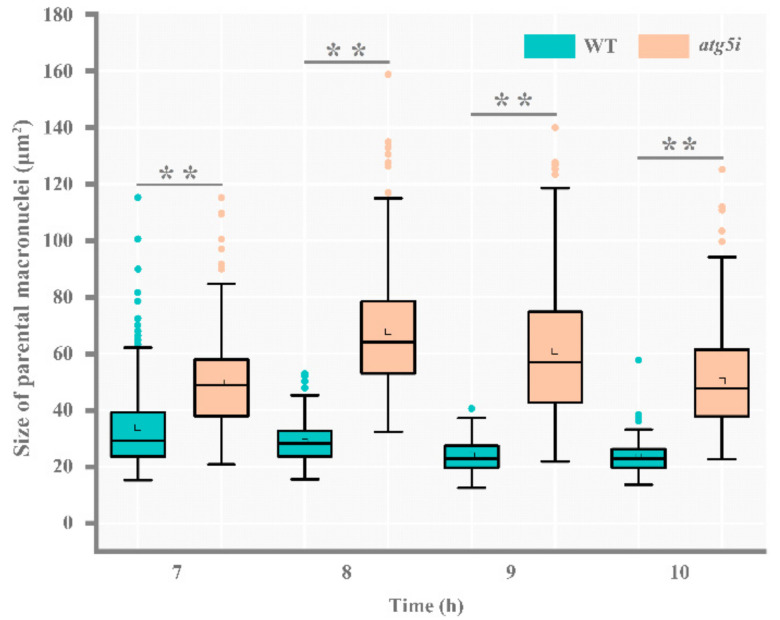
Condensation of paMAC was prevented after RNA interference of *ATG5*. Comparison of parental macronuclear size of mating WT cells and mating *atg5i* mutant cells at 7, 8, 9, and 10 h. Box plot explanation: upper horizontal line of box, 75th percentile; lower horizontal line of box, 25th percentile; horizontal bar within box, median; upper horizontal bar outside box, 90th percentile; lower horizontal bar outside box, 10th percentile. The green and orange circles represent the outliers of WT cells and *atg5i* mutant cells, respectively. *n* = 100 (cell number of pair cells). An unpaired sample *t* test was used for statistical analysis. ** *p* < 0.01.

MDC-labeled autophagosomes and LTR-labeled lysosomes gradually aggregated to the paMAC, and the entire paMAC was acidified in WT cells (Figure 4A(I–III)). The fluorescence intensities of LTR, MDA, and Hoechst in the acidified paMAC were similar (Figure 4B(a–c)). In the *atg5i* mutants, autophagosomes and lysosomes failed to aggregate near the paMAC, which maintained its blue fluorescence, indicating failure to acidify (Figure 4A(IV–VI),B(d–f)). Knockdown of *ATG5* hindered the fusion of the paMAC and autophagolysosomes. In addition, the delay developmental meiotic products were progressively acidified and degraded with the development of the paired cells (Figure 5). The paMAC was enlarged and loose during the late sexual-reproduction stage in the *atg5i* mutants. In WT cells, the median value of the paMAC area was 29.12 μm^2^ at 7 h and 22.92 μm^2^ at 10 h (*n* = 100). In *atg5i* mutants, the median was 48.8 μm^2^ at 7 h and 47.7 μm^2^ at 10 h (*n* = 100). The size of paMAC in *atg5i* mutant paired cells was significantly larger than that in WT paired cells (*p* < 0.01) (Figure 6). These results indicated that Atg5 was related to the pyknosis of the paMAC and mediated the acidification and degradation of paMAC.

### 3.3. Atg5 Mediated the PND of paMAC by Regulating the Function of Atg8.2

**Figure 7:**
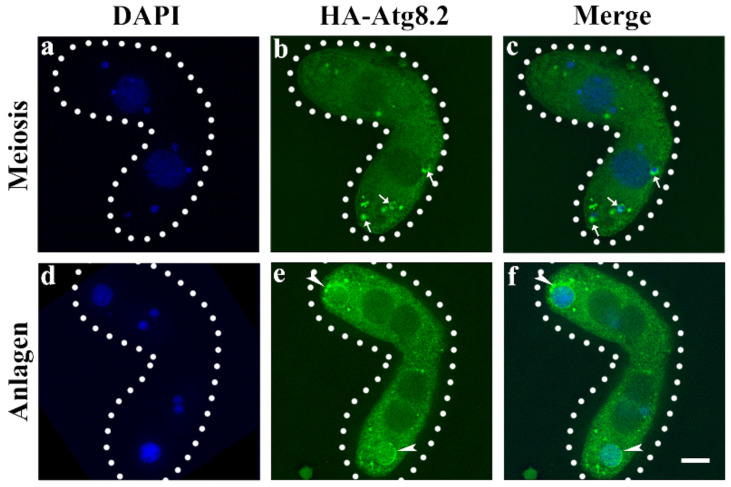
Localization of HA-Atg8.2 in the stage of meiosis and macronuclear anlagen. Cells collected at 5 h and 12 h after mixing were fixed and processed for immunofluorescence staining with anti-HA primary and FITC-conjugated secondary antibodies. Cellular nuclei were stained with DAPI to visualize DNA. (**a**,**d**), DAPI; (**b**,**e**), FITC; (**c**,**f**) Merge. The white arrows in (**b**) and (**c**) point to the meiosis products of MIC to be degraded, and the white arrows in (**e**,**f**) point to the paMAC to be degraded. Dashed circle represents the cell outline of *Tetrahymena*. Fluorescent images were taken with a DeltaVision deconvolution microscope. Scale bar, 10 μm.

**Figure 8:**
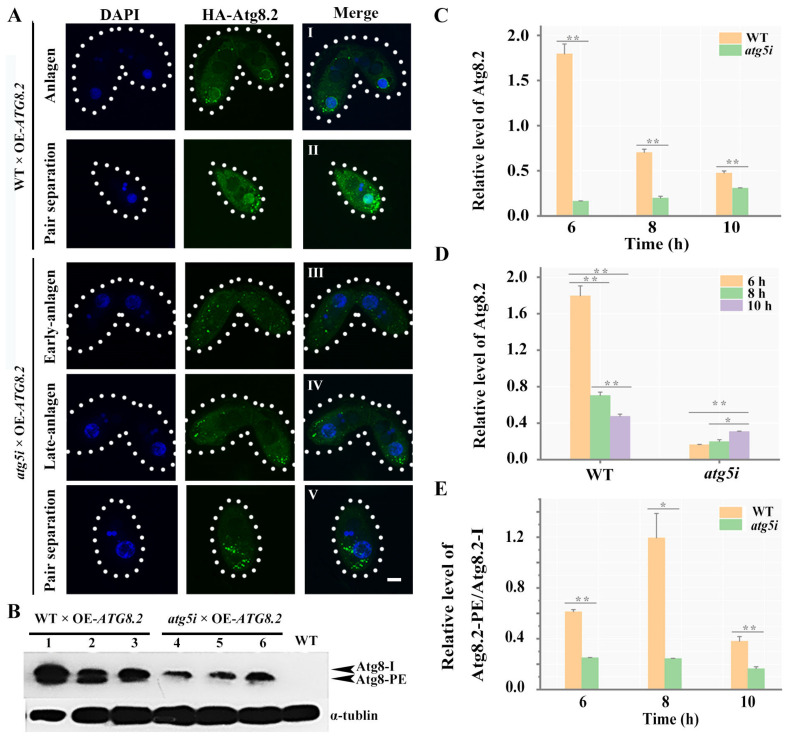
RNA interference of *ATG5* decreased the expression and lipidation of Atg8.2 and hindered the localization of Atg8.2 on the paMAC. (**A**): WT and *atg5i* mutant paired cells during sexual reproduction were used to analyze the subcellular localization of Atg8.2. I, WT mating cells at the anlagen stage; II and V, WT cell and *atg5i* mutant cell at the pair separation stage; III, *atg5i* mating mutant cells at the early-anlagen stage.; IV, *atg5i* mating mutant cells at the late-anlagen stage. Fluorescent images were taken with a DeltaVision deconvolution microscope. Dashed circle represents the cell outline of *Tetrahymena*. Scale, 10 μm; (**B**): The expression of Atg8.2 was detected by Western blot in WT and *atg5i* mutant paired cells. Protein samples were prepared at 6 h (lanes 1 and 4), 8 h (lanes 2 and 5), and 10 h (lanes 3 and 6) after mixing different mating types of cells; (**C**–**E**): Gray intensity analysis of the Western blot results was performed with Image J software. The unpaired sample *t* test was used in **C** and **E**, and a paired sample *t* test was used in (**D**). * *p* < 0.05 and ** *p* < 0.01.

Atg5 regulates the formation of Atg8-PE (phosphatidylethanolamine, PE) by forming a complex with Atg12 and Atg16 in yeast and mammalian cells [46]. Atg8.2 specifically regulates the fusion between autophagosomes and paMAC in *Tetrahymena* [34]. However, the homologs of Atg12 failed to be identified in *Tetrahymena.* To determine whether Atg5 mediated the PND of paMAC by regulating Atg8, we first constructed the HA-*ATG8.2* cell line (Figure S4) and observed the localization of Atg8.2 during the sexual-reproduction stage (Figure 7). Atg8.2 was localized in the cytoplasm during early sexual reproduction. When the paired cells developed to the stage of nuclear selection, Atg8.2 formed punctuated localization around the degraded meiotic products (Figure 7a–c). During the anlagen stage, Atg8.2 gradually aggregated to the paMAC and localized on the nuclear membrane of paMAC (Figure 7d–f). Then, HA-*ATG8.2* cells were mated with WT cells and *atg5i* mutants, and Cd^2+^ was added to induce the expression of *ATG8* and silencing of *ATG5*. In the paired cells of WT and HA-*ATG8.2*, Atg8.2 was first recruited to the vicinity of paMAC and was gradually localized to the paMAC during the PND stage (Figure 8A(I,II)). By contrast, in the paired cells of *atg5i* and HA-*ATG8.2*, Atg8.2 failed to localize to the paMAC normally (Figure 8A(III–V)). The expression level of Atg8.2 significantly decreased after *ATG5* knockdown (Figure 8B,C). Furthermore, the expression level of Atg8.2 gradually decreased with the development of paired cells during the PND stage in the paired cells of WT and HA-*ATG8.2*. Conversely, the expression level of Atg8.2 gradually increased after *ATG5* knockdown (Figure 8D). These results indicated that Atg5 was necessary for the localization of Atg8.2 on the paMAC and that *ATG5* knockdown led to the abnormal accumulation of Atg8.2. The ratio of Atg8.2-PE/Atg8.2-I significantly decreased during sexual reproduction in *atg5i* mutants (Figure 8E). *ATG5* knockdown prevented Atg8.2-PE formation. These results are consistent with the defective localization of Atg8 on the paMAC. Taken together, these results confirmed that Atg5 mediated the selective autophagy of the paMAC by regulating Atg8.2 function in *Tetrahymena*.

## 4. Discussion

Nuclear autophagy occurs in yeast and many eukaryotes, including unicellular organisms and mammals [15]. The PND of paMAC during the sexual reproduction of *Tetrahymena* provides an ideal model for studying the regulation of nuclear autophagy. Atg5 was initially localized in the cytoplasm during the sexual reproduction of *Tetrahymena*. At the early anlagen stage, Atg5 was localized on the paMAC when the paMAC was still in the anterior or middle of the cell. The localization pattern of Atg5 on the paMAC was consistent with that of Atg8.2 [34]. However, their localization was earlier than that of Atg4.1 on the paMAC [35], indicating that these autophagy proteins had already begun to function at the initial stage of the PND of paMAC. The paMAC failed to migrate from the anterior or middle of the cytoplasm to the posterior region in the Atg8- or Vps34-deficient mutants [31,34]. Atg8 and Vps34 were used to position paMAC besides performing the autophagic/lysosomal pathway. Atg8 and Vps34 played collaborative or sequential roles in the PND stage [31]. We also found that the migration of paMAC was inhibited in *atg5i* mutants. Atg5 regulated the lipidation of Atg8 and mediated the degradation of the paMAC.

In the *atg5i* mutants, the degradation of meiotic products was delayed, but they eventually acidified and degraded. In mammalian autophagy, an *ATG5/ATG7*-independent alternative pathway exists, and neither the covalent combination between Atg5 and Atg12 nor the LC3 (LC3-I) to PE-conjugated LC3-II occurs in the alternative pathway [47]. The phenomenon also exists in mitophagy, which can be regulated by different pathways. Type 1 mitophagy depends on beclin1 and PI3K, whereas Type 2 mitophagy is independent of beclin 1 and PI3K. Type 3 mitophagy (micromitophagy) forms mitochondrion-derived vesicles (MDV) [48,49,50]. MDV formation and transit to lysosomes occur independently of the autophagic proteins Atg5 and LC3 [51]. Atg8 is involved in regulating the degradation of meiotic products in *Tetrahymena* [34]. Herein, *ATG5* knockdown significantly reduced the expression and lipidation of Atg8.2, but meiotic products were still acidified and degraded. This finding suggested that the degradation of meiosis products may have an *ATG5/ATG7*-independent alternative autophagy pathway.

**Figure 9:**
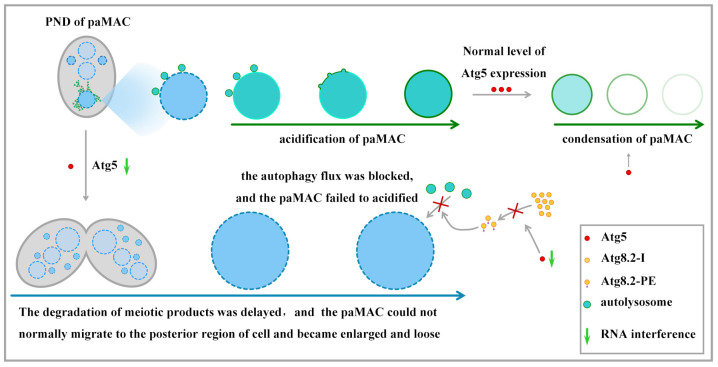
Cartoon illustrating the role of Atg5 during PND in *Tetrahymena*.

Atg8 family members are important for autophagosome-lysosome fusion by recruiting PLEKHM1 to autophagosome [52,53]. GABARAP protein, a member of the Atg8 family, mediates autophagosome-lysosome fusion by regulating the lipid composition of autophagosomes [54]. The *Caenorhabditis elegans* Atg8 homologs interact with the HOPS tethering complex to promote the fusion of autophagosomes with lysosomes [55]. Atg8 dissociates from the external membrane of autophagosomes through the cysteine protease Atg4 to achieve recycling [56]. Accordingly, the spatial limitation of Atg4 impedes autophagosome–vacuole fusion in yeast, and Atg8 is removed from autophagosomes before successful fusion [57]. Atg8 promotes the recruitment of tethers and other proteins in the process of autophagosome-lysosome fusion. However, after completing this mission, they have to be completely removed to allow subsequent fusion [58]. In *Tetrahymena*, the acidification of paMAC involves the membrane fusion of autolysosome and paMAC. The paMAC does not acidify until it is completely labeled with Atg8.2 on the periphery of paMAC, and the signal of Atg8.2 can still be detected on the paMAC when acidification occurs [34]. Herein, Atg8.2 was involved in regulating the membrane fusion between paMAC and autolysosome. The lipidation of Atg8 was blocked by knocking down *ATG5*, and the acidification of paMAC failed to occur (Figure 9). These results indicated that Atg5 mediates the Atg8 lipidation conjugation system and is required for membrane fusion between the paMAC and autolysosome. In mammalian macroautophagy, Atg5-Atg12 complexes are recruited by the PI3P-binding protein Tectonin beta-propeller repeat-containing 1 localized on lysosomes/autolysosomes to facilitate autophagosome maturation and autophagosome-lysosome fusion [59]. In *Tetrahymena*, Atg5 directly participates in the regulation of the fusion of autolysosomes and paMAC.

The paMAC migrates from the anterior or middle of the cytoplasm to the posterior region, condenses, gradually acidifies, and degrades during the PND of WT cells. By contrast, the paMAC enlarges, loosens, and fails to degrade in the *atg5i* mutants. The lipidation of Atg8.2 and its localization on the paMAC were inhibited after knocking down *ATG5* (Figure 9). Therefore, Atg5 is involved in the regulation of the selective autophagy of paMAC. Atg5 is necessary for the expression and lipidation of Atg8, as well as the acidification and degradation of paMAC. Atg5 regulates the acidification and degradation of paMAC by mediating the lipidation of Atg8 in *Tetrahymena*.

## Data Availability

Not applicable.

## Supplementary Materials

The following are available online at https://www.mdpi.com/2073-4409/10/11/3071/s1, Table S1: Primers used in the study, Figure S1: Localization of HA-Atg5 during the early stage of sexual reproduction of *Tetrahymena thermophila*. Cells collected after 2 and 4 h of mixing were fixed and processed for immunofluorescence staining with anti-HA primary and FITC-conjugated secondary antibodies. Cellular nuclei were stained with DAPI to visualize DNA. Fluorescent images were taken with a DeltaVision deconvolution microscope. Scale bar, 10 μm, Figure S2: Construction and identification of *Tetrahymena* strains overexpressing *ATG5*. A: diagram of the *ATG5* construct at the *MTT1* locus. B: PCR identification of *ATG5*-overexpressing mutants. Arrowheads indicate positive recombinant fragments (1.5 Kb) and WT fragments (0.7 Kb). M: Trans 2K plus DNA marker; WT: wild type; OE-*ATG5*-B/C: *ATG5*-overexpressing mutants of B2086 or CU428; C: expression level of *ATG5* was analyzed by qRT-PCR. Total RNA was isolated from conjugating cells after mixing for 8 h, Figure S3: Construction of *ATG5* RNAi knockdown cell lines and Western blot analysis. A: diagram of the *ATG5* RNAi knockdown construct and the wild-type *ATG5* locus. B: The expression levels of HA-Atg5 protein in conjugated cells were detected using Western blot. α-Tublin served as the internal reference. Lanes 1: Wild type; Lanes 2–4: Samples were collected at 6, 8, and 10 h after wild-type cells were paired with the OE-*ATG5* mutants; Lanes 5–7: Samples were collected at 6, 8, and 10 h after *atg5i* mutant cells were paired with the OE-*ATG5* mutant cells, Figure S4: Construction and identification of *Tetrahymena* strains overexpressing *ATG8.2*. A: diagram of the *ATG8.2* construct at the *MTT1* locus. B: PCR identification of *ATG8.2* overexpressing mutants. Arrowheads indicate positive recombinant fragments (0.85 Kb) and WT fragments (0.7 Kb). M: Trans 2K plus DNA marker; WT: wild type.
