# Correction: Bo et al. Atg5 Regulates Selective Autophagy of the Parental Macronucleus during *Tetrahymena* Sexual Reproduction. *Cells* 2021, *10*, 3071

**DOI:** 10.3390/cells13151305

**Published:** 2024-08-05

**Authors:** Tao Bo, Yu Kang, Ya Liu, Jing Xu, Wei Wang

**Affiliations:** 1 Key Laboratory of Chemical Biology and Molecular Engineering of Ministry of Education, Institute of Biotechnology, Shanxi University, Taiyuan 030006, China; botao@sxu.edu.cn (T.B.); 201923002007@email.sxu.edu.cn (Y.K.); 201723002012@email.sxu.edu.cn (Y.L.); xujing@sxu.edu.cn (J.X.); 2 College of Life Sciences, Shanxi University, Taiyuan 030006, China

In the original publication [1], there was a mistake in Figure 2 as published. The merged images originated from digital images acquired utilizing a Delta Vision Elite deconvolution microscope. The amalgamated images were generated by superimposing distinct layers, resulting in variations in layer signals compared to DAPI staining. Owing to elevated background levels, certain layer signals were obscured, while others emerged through the superimposition of different layer signals. The correct Figure 2 appears below. The authors state that the scientific conclusions are unaffected. This correction was approved by the Academic Editor. The original publication has also been updated.

## Figures and Tables

**Figure 2:**
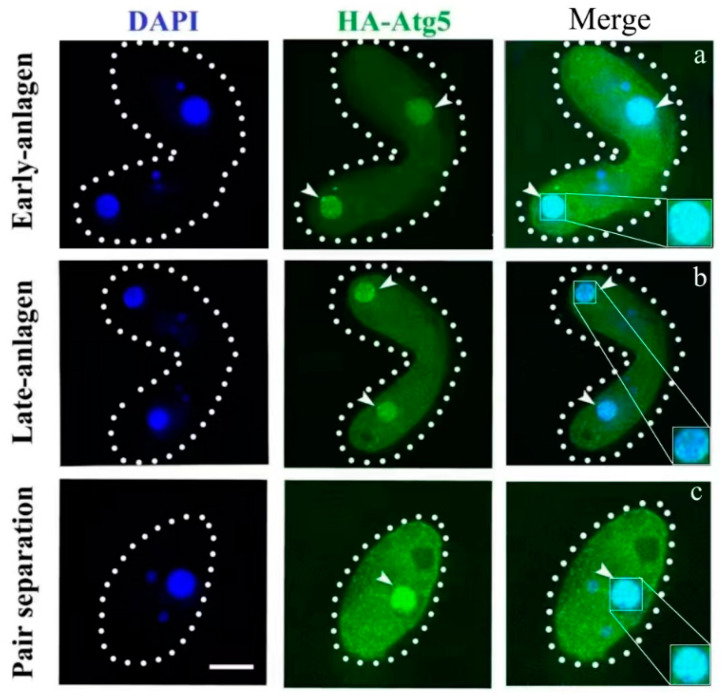
Localization of HA-Atg5 during the sexual reproduction of *Tetrahymena thermophila*. Cells collected at 6, 8, and 12 h after mixing were fixed and processed for immunofluorescence staining with anti-HA primary and FITC-conjugated secondary antibodies. Cellular nuclei were stained with DAPI to visualize DNA. (**a**) cells at the early-anlagen stage; (**b**) cells at the late-anlagen stage; (**c**) cells at the pair separation stage. Dashed circle represents the cell outline of *Tetrahymena*. The white arrows point to the paMAC to be degraded. The white box shows a sharp enlargement of the paMAC. Fluorescent images were taken with a DeltaVision deconvolution microscope. Scale bar, 10 µm.
