# Supplementary material for: Atg5 Regulates Selective Autophagy of the Parental Macronucleus during Tetrahymena Sexual Reproduction
Source: Cells. 2021 Nov 8;10(11):3071. doi: 10.3390/cells10113071 (PMC8623110; doi:10.3390/cells10113071)
Supplement: Supplementary file 1 [file cells-10-03071-s001.zip › Supplementary Materials.pdf]

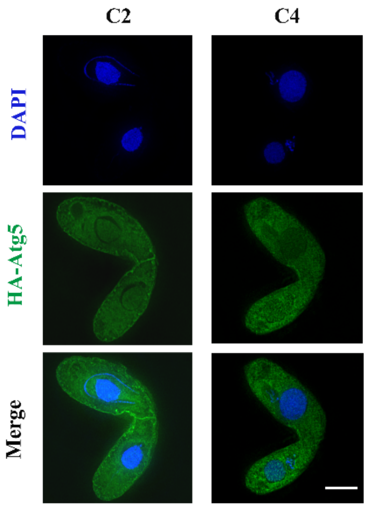

Figure S1 Localization of HA-Atg5 during the early stage of sexual reproduction of *Tetrahymena thermophila.* Cells collected after 2 and 4 h of mixing were fixed and processed for immunofluorescence staining with anti-HA primary and FITC-conjugated secondary antibodies. Cellular nuclei were stained with DAPI to visualize DNA. Fluorescent images were taken with a DeltaVision deconvolution microscope. Scale bar, 10 μm.

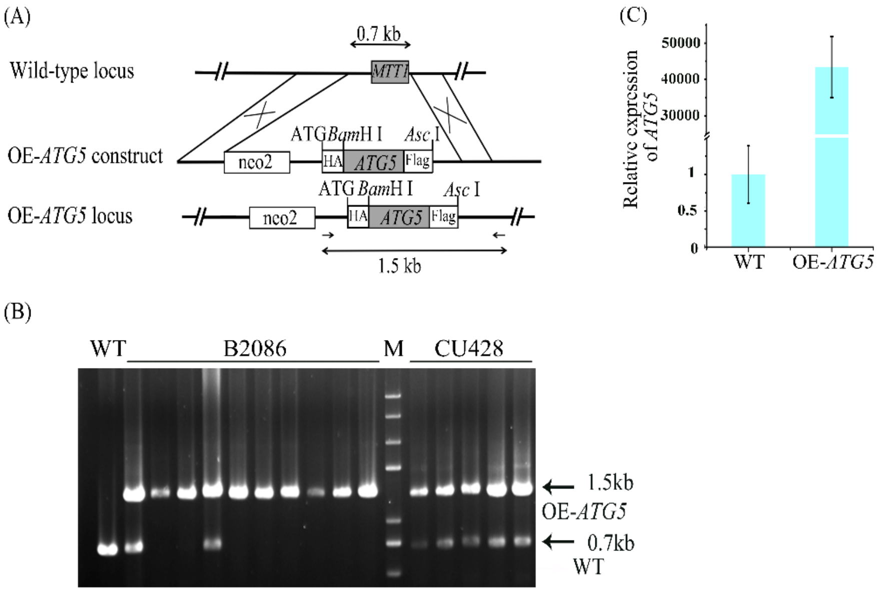

Figure S2 Construction and identification of *Tetrahymena* strains overexpressing *ATG5.* A: diagram of the *ATG5* construct at the *MTT1* locus. B: PCR identification of *ATG5*-overexpressing mutants. Arrowheads indicate positive recombinant fragments (1.5 Kb) and WT fragments (0.7 Kb), respectively. M: Trans 2K plus DNA marker;

WT: wild type; OE-*ATG5*-B/C: *ATG5*-overexpressing mutants of B2086 or CU428; C: expression level of *ATG5* was analyzed by qRT-PCR. Total RNA was isolated from conjugating cells after mixing for 8 h.

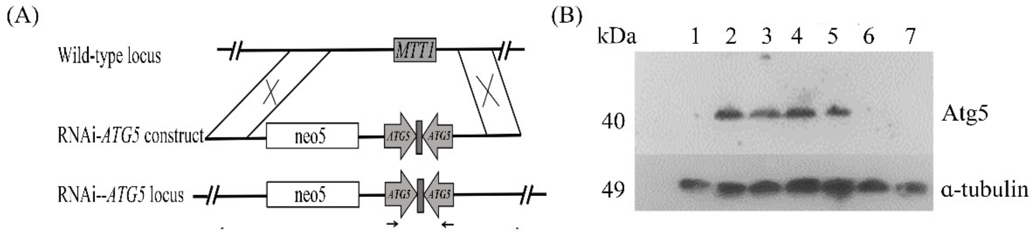

Figure S3 Construction of *ATG5* RNAi knockdown cell lines and Western blot analysis. A: diagram of the *ATG5* RNAi knockdown construct and the wild-type *ATG5* locus. B: The expression levels of HA-Atg5 protein in conjugated cells were detected using Western blot. α-Tublin served as the internal reference. Lanes 1:Wild type; Lanes 2–4: Samples were collected at 6, 8, and 10 h after wild-type cells were paired with the OE-*ATG5* mutants; Lanes 5-7: Samples were collected at 6, 8, and 10 h after *atg5i* mutants were paired with the OE-*ATG5* mutants.

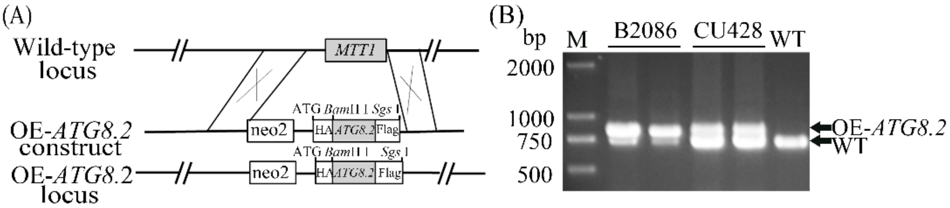

Figure S4 Construction and identification of *Tetrahymena* strains overexpressing *ATG8.2*. A: diagram of the *ATG8.2* construct at the *MTT1* locus. B: PCR identification of *ATG8.2* overexpressing mutants. Arrowheads indicate positive recombinant fragments (0.85 Kb) and WT fragments (0.7 Kb), respectively. M: Trans 2K plus DNA marker; WT: wild type.

Table S1 Primers used in the study

Primer name Sequence（ 5'→3'）
OE-*ATG5*-F: GGATCC GCAGAGGAACAAAAATAAACAGATTTTG
OE-*ATG5*-R: GGCGCGCCTTTTTATATACATAGATAAGTGAAGCCAT
RNAi-*ATG5*-3'F: CTGCAGTAGGACCCATCTATGAAAGGAGAAT

RNAi-*ATG5*-3'R: CCCGGGGATGAGAGAGTTATATACGACCTATAT

RNAi-*ATG5*-5'F: GGATCCTAGGACCCATCTATGAAAGGAGAAT

RNAi-*ATG5*-5'R: GTTTAAACGATGAGAGAGTTATATACGACCTATAT

MTT1-F: GCTACGTGATTCACGATTTATGCAATG

MTT1-R: CGAAACTGATTTTATGCAATTATGAATTAC

OE- *ATG8.2*-F: GGATCC ATGGACGCTCAAAATTATAAACCCTT

OE- *ATG8.2*-R: GGCGCGCC TCAAATTGATCCAAATACTTCTTGATC

Underlines indicate restriction endonuclease sites: *BamH* I: GGATCC，*Sgs* I: GGCGCGCC，

Pst I: CTGCAG，*Sma* I: CCCGGG，*Pme* I: GTTTAAAC．RNAi for RNA interference
